# Book Review

**Published:** 1911-09

**Authors:** 


**Outlines of Zoology.** By J. Arthur Thomson, M.A. Fifth Edition. Pp. xxi., 855. London: Oxford Medical Publications. 1910.—We are delighted to see the new edition of this valuable textbook. It has a charm which is peculiarly its own, and by reason of this stands out prominently among textbooks as the most suitable to put into a beginner's hands. By this we do not mean that the book is suited only for a beginner's needs, for by the judicious use of different type the author has succeeded in cramming inside its covers more information than is to be found in many a more pretentious book, thus making it a fit guide and companion to more advanced students both in the laboratory and in the museum. In one feature—illustrations—this edition is decidedly superior to its predecessor, not only in quality but also in number. New figures have been added, old figures improved, but some old friends have disappeared. They may have appeared useless, but still they helped many a student over a difficult point. In addition to a thorough revision of the general portion, numerous groups have received a fuller treatment, *e.g.* Protozoa, Cœlenterata, etc. Several of the less well-known forms—we mention a few only—Phoronis, Pycnogonum and Cephalodiscus, have been treated more fully, and so adequately illustrated by diagrams that they represent now more than mere names to the student. The last, though in our opinion not the least important, feature is that the author has begun to treat the vertebrates on a slightly more generous scale, although he has not yet given the amount of space and the number of illustrations that are necessary if the textbook is indeed to be a " manual which students of zoology may use in the lecture room, museum and laboratory."

**A Manual of Surgical Anatomy.** By Charles R. Whittaker, F.R.C.S. (Ed.). Pp. x., 248. Edinburgh: E. & S. Livingstone. 1910.—The author tells us in his preface that this work is designed to give the main outlines of his subject. This object he has certainly attained. Although condensed into less than 250 small pages, the essentials of surgical anatomy are presented in a clear and readable form. For its size it covers a great deal of ground, and is thoroughly up to date, as can be seen by the devotion of nearly a page to the parathyroid glands. We are pleased to see that the lymphatic system, so often neglected in works of a like nature, receives its due attention, although perhaps in the case of the lymphatics of the mammary gland a little more detail might be useful. The illustrations, though mainly diagrammatic, are clear and instructive. We can recommend this book to the senior student preparing for his final.

**Transactions of the College of Physicians of Philadelphia.** Vol. xxxi. Philadelphia: W. J. Dornan. 1909.—A well-printed volume of 750 pages containing the papers read during the year must be of value not only as a reminder to those who heard the reading of the papers, but to a much larger body of readers to whom they are new. Dr. James Tyson, the President, regrets that the volume is not further enlarged by the inclusion of papers read before the sections, but that the absence of sufficient funds makes their inclusion impossible. A noteworthy portion of the volume consists of the memoirs of recently deceased Fellows of the College—John Hooker Packard, M.D., William Thomson, M.D., Charles Stewart Wurts, M.D., and Horace Y. Evans, M.D. As one of the biographers remarks of the subject of his memoir, " We are all broader men for having known and loved him. Some of us wonder that we did not better understand his silent influence when here.

" Soft, scentless, feathery flowers of blue
Were in my garden border set;
Beside them, clothed in tender green,
Grew the dear, fragrant mignonette.

"I plucked a spray of blossoms blue,
  And kept them with me in my room.
And suddenly I 'came aware
  Of faintest fragrance from their bloom!

"I could not think what it might mean,
  That such a fragrance I could get
From scentless flowers, until I thought—
  They grew beside the mignonette!"

**The Medical Annual (1910)** has advocated the use of injection of sea-water, as advised by Dr. Robert Simon, of Paris; here also we find a paper by Dr. Theodore de Boutillier on the internal use of sea-water by subcutaneous injection, from which we learn that the practice was suggested by the universally-accepted theory of the marine origin of life, supported by the chemical fact that sea-water and blood plasma contain the same minerals in almost identical proportions. An aseptic isotonic, sea-water has been shown to act very differently from a physiological salt solution, and has given the following results: "Increase of appetite, improvement in cases of constipation, marked improvement in cases of diarrhœa, increase in the quantity of urine eliminated, improvement in insomnia, and relief of functional dysmenorrhœa. In cases of malnutrition, and in some cases of tuberculosis, the gain in weight has been marked. In cases of pulmonary tuberculosis, with cough and expectoration, it has been found that the expectoration ceased, and within a few weeks the cough subsided." We cannot but think that fallacies in observation, or insufficiency of the same, must account for some of these results, and that the method by suggestion or even faith-healing may compete with sea-water to the advantage of the spiritualistic methods. Dr. Martin H. Fischer's prize essay on "The Problem of Œdema" should be studied by all pathologists. It occupies two hundred pages of the book.

**The Medical Annual (1911).** Bristol: John Wright & Sons. 1911.—The twenty-ninth issue of this hardy annual will maintain the reputation of the series. The publishers may well "watch with parental pride the growth of the book, and the increasing hold it has gained upon the affection and confidence of the medical profession in all parts of the world." The personnel of the editorial staff remains much the same, but we notice a slight increase in the list of contributors, although the size of the volume does not increase. The editor's table, and the list of asylums, hospitals, licensed houses, sanatoria, nursing and other homes are very useful, as also are the very numerous advertisements. The busy general practitioner must be grateful for the annually revised lists of such institutions. We notice that various drug firms are encouraging the use of what they call Sea-Water Plasma, and it is satisfactory to learn that in no single case have there been *ill results* from the injection of diluted sea-water, *alias* isotonic Sea-Water Plasma.

**Medical Guide to German, Austrian, and Swiss Watering Places and Sanatoria.** Edited by Harold Morré, M.D. Second Edition. Berlin: Eugen Schachtel. 1910.—This booklet is practically a list of many spas, with a table of indications as to the suitability of particular spas for particular diseases. We notice that Arosa is the place for atheromatose, Godesberg for chorea, Ahlbeck for epilepsy, and Dürkheim for leukæmia, etc. The numerous advertisements are quite the most essential part of the book.

**The Occurrence of Infantile Paralysis in Massachusetts in 1908.** Reported for the Massachusetts State Board of Health by Robert W. Levett, M.D., and Herbert C. Emersen, M.D. **Infantile Paralysis in Massachusetts in 1909.** (Reprint.) Boston: Wright & Potter Printing Co. 1909.—The epidemic wave of cerebro-spinal meningitis which started in Scandinavia visited the northern portion of the United States in 1908 and 1909. These two reports of the Massachusetts State Board of Health are models of modern methods of research applied to the investigation of an epidemic disease of which but little is known. The reports confirm the findings of recent observers on many important points, such as the infectious nature of the complaint, its low power of contagion, and its origin from a virus so small as to pass through the porcelain filter and to escape detection by the modern microscope. Evidence is brought forward to show that the organism probably gains entrance by the mouth, and emphasis is laid on the diagnostic value of dyspeptic symptoms early in the disease. Lucas's analysis of the prodromal symptoms, and the results of his examination of the blood and cerebro-spinal fluid in the early stages of the disease, are of the highest value, for if any benefit is to be obtained by treatment it is essential that the disease be diagnosed before paralysis sets in. The eight types of the disease are described, and details given of so-called " abortive " cases. Methods of treatment, both medical and surgical, are fully discussed. These reports not only summarise our present-day knowledge of this disease, but they also abound in suggestion for future lines of investigation.

**Hæmoglobinuria.** By Ambrose E. L. Charpentier, M.D. Pp. vii., 111. London: Baillière, Tindall & Cox. 1910.—The preface gives us no clue as to the author's reasons for publishing this compilation in the form of a separate book rather than as a contribution to a medical journal. He does not pretend to any original views, being content with a comprehensive survey of the literature of the various forms of hæmoglobinuria, with fairly complete records of several cases which have been observed by him. Those who are looking into the subject will be glad of the long list of references to the literature with which the book closes.

**A Handbook of Intestinal Surgery.** By LEONARD A. BIDWELL, F.R.C.S. Second Edition. Pp. xiv., 215. London: Baillière, Tindall and Cox. 1910.—We have here presented in small compass a well-written and clear description of a great variety of intestinal sutures with numerous illustrations. Some useful hints for the teaching of intestinal surgery in class are also given. Some account is included of the principal operations on the stomach, intestines and appendix. It will be noticed that the author is at great pains to limit to the utmost the indications for colostomy. The book concludes with two chapters on the preparation of the patient and after-treatment, not only in hospitals but also in private houses. For those who do not care to keep by them the large textbooks of operative surgery, but who require to refresh their memories of the intricacies of intestinal methods, Mr. Bidwell's monograph will be very valuable.

**Pye's Elementary Bandaging and Surgical Dressing.** Twelfth Edition. Revised and partly rewritten by W. H. CLAYTON-GREENE, M.B., assisted by V. ZACHARY COPE, M.D. Pp. viii., 235. Bristol: John Wright & Sons Ltd. 1910.—Concerning this well-known and valuable little pocket-book, it need only be said that it has been brought up to date by the inclusion of some account of Bier's treatment, Michel's clips, etc. It ought to be purchased and learnt by heart by every out-patient dresser.

**An Introduction to Surgery.** By RUTHERFORD MORISON, F.R.C.S. Bristol: John Wright & Sons Ltd. 1910.—This little book of 155 pages has the merit of being something quite unusual. Its distinguished author has resolutely set himself the very narrow limits of a few general principles, and these are dealt with in the briefest possible manner. The illustrations, on the other hand, which are mostly provided by Mr. Pybus, the Surgical Registrar to the Newcastle Infirmary, are lavish, artistic, and exceedingly well chosen. The text, which only professes to be an abstract of Mr. Morison's lectures, is thus condensed, scrappy and uninteresting, whilst the pictures form the most fascinating presentment of the subject that it is possible to imagine. The following are the subjects treated: Inflammation, bacteria, ulcers, gangrene, syphilis, tubercle, malignant disease, hæmophilia, wounds, some affections of the abdominal viscera, the indications for operation and the X-rays.

**Hernia: its Cause and Treatment.** By R. W. MURRAY, F.R.C.S. Pp. vi, 184. London: J. & A. Churchill. 1910.—This short and readable monograph strives to teach one main point, namely that for all practical purposes all hernias take place into preformed sacs. The author follows Hamilton Russell, of Melbourne. He argues that a truss cannot cure a hernia, and that there is always left a potential sac. This also applies to direct herniæ, which he thinks start in a congenital pouch, the same being true of femoral, sciatic and umbilical. With regard to infantile hernia, he quotes Russell as saying that the sac is a perfectly normal funicular process, the sac in front being an elongation of the tunica vaginalis owing to its getting " hung up " as it and testis descend. Following on these conclusions, he attributes his success in the radical cure to very complete removal of the sac, and not to any special method of dealing with the fascia and muscles beyond bringing them together with sutures. The patients are usually allowed up on the tenth day, and can get to work in about three weeks.

**Enlargement of the Prostate.** By C. Mansell Moullin, M.D. Oxon. Fourth Edition. Pp. ix., 176. London : H. K. Lewis. 1911.—To those interested in the surgery of the prostate we commend this excellent manual, based on the personal experience of the writer with a wide knowledge of the subject expressed in a clear manner. The whole range of the normal and pathological states is dealt with, and we may refer to a few points in treatment. For the suprapubic operation the author employs a transverse incision, and he speaks favourably of the perineal operation, which is so little practised in England, and also to some extent of the Bottini operation with the cautery. He advises that all cases should have preliminary preparation before a radical operation is done, and a short chapter on the choice of treatment may be read with advantage. An ideal mortality of only 5 per cent. should be aimed at, which is only likely if the bladder and kidneys have not already been damaged beyond repair by the indiscriminate employment of catheters.

**Le Village Sanatorium.** Paris : L'Enseignement Médico-Mutual International. 1910.—This contribution to the study of sanatoria by an architect, H. G. Richter, is prefaced by the Countess of Aberdeen, to whom the booklet is dedicated. As a result of the conjoined efforts of the physician and the architect, we have now established the Sanatorium Hotel, the Sanatorium École, the Sanatorium Maison de Santé, the Sanatorium Maison de Campagne. The author wishes to add, and proposes plans for, Le Village Sanatorium, and further Le Village des Guéris.

**Physiological Principles in Treatment.** By W. Langdon Brown, M.A., M.D., F.R.C.P. 2nd Edition. Pp. vii, 392. London : Baillière, Tindall & Cox. 1910.—We are glad to see that a second edition of this excellent little book has been called for in the short space of two years, as it appears to us a happy augury for the future of medical treatment, and a reassuring index of the interest in the scientific principles which should underlie it, among the practitioners and senior students of medicine. The second edition has been brought up to date by the inclusion of new work on the movements of the stomach, endogenous purins, auto-intoxication from the intestine and irregular cardiac rhythms, and on various other minor details. The index has also been extended, which we regard as a very valuable improvement. In reviewing the first edition we congratulated both the author on his book and the practitioner on having the opportunity of reading so excellent a treatise, and we cannot do more than say that while all those who have read the first edition will not hesitate to buy and read the second without any words of advice from us, to those who omitted to acquire the older volume an opportunity is now given of obtaining it in a distinctly improved form which we hope they will on no account neglect. We sincerely hope that the second edition of this useful and eminently readable book will be exhausted in a still shorter period of time than the first.

**Studies on Thyroid.** I. *The Relation of Iodine to the Physiological Activity of Thyroid Preparations.* By Reid Hunt and Atherton Seidell. Pp. 115. Washington: Government Printing Office. 1909.—This pamphlet forms No. 47 of the valuable bulletins published by the Hygienic Laboratory of the Public Health and Marine Hospital Service of the United States, and is characterised by the same detailed methods of experiment and description which are found in the other numbers of this useful series of publications. Following on a succinct and lucid historical introduction, the authors point out that some investigators hold that the activity of the thyroid is dependent on and directly proportional to the amount of iodine it contains. Others deny this relationship entirely, and believe that the iodine content is unimportant, while a third group believe that though iodine content and physiological activity vary concomitantly, the former is not causally related to the latter. After a discussion of the evidence for these views, they go on to explain their methods for testing the claims of iodine to be regarded as causally related to the physiological activity of the thyroid. These consisted of observing the effect of iodine-containing thyroids and others containing no iodine on the resistance of animals to certain poisons. A number of animals of different kinds were used, and the experiments were varied in all sorts of ways: animals were fed with various iodine preparations, and the amount of iodine in the thyroid thereby increased. The authors show that resistance to certain poisons such as acetonitrile and ethylene cyanide is increased, whereas to morphine, heroine, codeine and chloral-hydrate it is diminished. In the case of a large number of poisons no effect was noted. Iodine-free thyroid was shown to be less potent in its action than iodine-containing thyroid. In Part II the authors discuss the nature of the relation between iodine percentage and the physiological activity of the thyroid, which they consider as the result of their experiments to be a causal one. Animals fed for a short time with iodoform or potassium iodide show an increased iodine content in their thyroids, which are also more active is producing resistance to acetonitrile when given to other animals. Full details of all the experimental work are given by the authors, and the system of controls seems to have been very thoroughly carried out.

**Uric Acid in the Clinic.** By ALEXANDER HAIG, M.A., M.D., F.R.C.P., assisted by K. G. HAIG. Pp. 306. London: J. & A. Churchill. 1910.—This book consists of clinical notes of nearly 400 cases of various conditions considered in the light of the author's well-known views on the subject of uric acid. It is, he tells us, a clinical appendix to his larger work, showing the basis on which his theories were formed and elaborated. How many are the ailments with which in his opinion the "uric acid" factor is connected may be seen from the list of diagnoses, which range through "headache," epilepsy, neurasthenia, "mental depression," asthma, chlorosis, "dyspepsia with weak heart," chorea, neuritis and neuralgia, Bright's disease, glycosuria and diabetes, "high blood pressure," Graves's disease, chronic and acute arthritis, bronchitis, and dermatitis, together with a group of seven cases labelled "abdominal" and six others "miscellaneous," among which, however, we have *not* found enlargement of the prepatellar bursa. In all cases with remarkable ingenuity and persistence the author traces the symptoms to the presence of uric acid, and his invariable prescription is a uric acid free diet, or speaking generally the exclusion of meat. Although the purine bodies may advantageously in certain cases be diminished or excluded altogether from the diet, we cannot help feeling that the author of this volume proves too much, and that the theory he holds is not always built upon the clinical observations, but rather that the latter are merely interpreted in the light of his preconceived opinions. For instance, he regards chlorosis occurring in young girls as due to the circulation of "colloid uric acid" in the blood, brought about by the consumption of meat and tea. However, a "uric acid free" diet is of no avail in clearing up the condition, but iron salts being "retentives"—that is, forming insoluble compounds with uric acid—do good by preventing its circulation in "colloid" form. In spite of many examples of this kind of reasoning, the book, like all collections of notes from actual cases, forms interesting reading, and with due reservations instructive reading also. There is a good index, and a short preliminary chapter intended for those who have not read the author's larger work.

**Practical Guide to the Diseases of the Throat, Nose and Ear.** By WILLIAM LAMB, M.D., C.M. Second Edition. Pp. xvi, 322. London: Baillière, Tindall and Cox. 1909.—A few years ago we favourably noticed the first edition of this work. In the present edition the sections on diagnosis have been expanded and new sections on treatment added. The author's style is interesting and lucid, the subject-matter is dealt with in an eminently practical manner, and the illustrations are good and sufficient. We know of no better manual to place in the hands of those beginning the study of diseases of the throat, nose and ear.

**Operations on the Ear.** By B. HEINE. Translated from the Second German Edition by W. LOMBARD MURPHY, M.B., B.C. Pp. xi, 204. London: Baillière, Tindall and Cox. 1908.—In Vol. XXII (1904) of this *Journal* we reviewed the first German edition of this book, and expressed the very favourable opinions we had formed of its merits. In the present translation of the second edition the chapters on Labyrinthine Suppuration and Meningitis have been rewritten by the author, and many other sections considerably expanded. The book is a most readable one. Not only is the technique of the usual aural operation well described, but the indications for the various procedures, the after-treatment, the results to be expected, and the possible dangers are fully and judiciously considered. For example, the sections on Ligature of the Jugular Vein, and on the Indications for Operation upon the Labyrinth, and for Operation in Meningitis, are very helpful, and are obviously the outcome of a careful study of the problems at issue in the light of experience gained in the large clinic of Professor Lucae. We should have liked some illustrations of the technique of labyrinthotomy, and we hope in a future edition to find the researches of Bárány discussed in greater fulness. The work has been excellently translated, and deserves the careful attention of all surgeons likely to be called upon to operate for aural diseases and their complications.

**Otitic Cerebellar Abscess.** By Dr. HEINRICH NEUMANN. Translated by RICHARD LAKE, F.R.C.S. Pp. 156. London: H. K. Lewis. 1909.—Dr. Neumann has collected 336 cases of temporal abscess and 196 of cerebellar abscess from the literature of the subject and from the records of Professor Politzer's clinic, all the cases having occurred since the year 1900. In the second part of the book an abstract of the histories of

165 cases of cerebellar abscess is given. In the first part the subject-matter is divided into sections on Statistics, Ætiology, Pathological Anatomy, Symptomatology, Diagnosis and Operative Treatment. As the author says, the diagnosis of otitic cerebellar abscess presents as a rule great difficulty. The treatment is also difficult. The latter fact is well proved by the writer's statistics, which show that only about one-fourth of the recorded cases were treated successfully. We consider this monograph to be a valuable contribution to the literature of the subject, and we are glad it has been made accessible to English readers through Mr. Lake's excellent translation.

**Hay Fever and Paroxysmal Sneezing.** By Eugene S. Yonge, M.D. Pp. 150. Edinburgh: William Green & Sons. 1910.—The author divides his work into three parts: (1) Hay Fever (Hay Asthma), (2) Paroxysmal Sneezing (Vasomotor Rhinitis), and (3) Idiopathic Rhinorrhœa (Nasal Hydrorrhœa), giving a very complete résumé of the contributions of various observers to our knowledge of these subjects. It may appear a difficult matter to make interesting reading from an historical survey of sneezing, but the author has succeeded here quite as well as in the more practical sections on ætiology, diagnosis and treatment. It is a book to be commended for those who want to know all about these troublesome complaints, and the therapeutic measures that are employed for their relief.

**A Textbook of Public Health.** By John Glaister, M.D., D.P.H. Second Edition. Pp. xxx, 622. Edinburgh; E. & S. Livingstone. 1910.—This textbook, which has been expanded from the second part of the author's former *Textbook of Medical Jurisprudence, Toxicology and Public Health,* and is now issued separately, is a characteristic student's handbook, and contains a great deal of sound information, which will, if duly assimilated, give the student a fairly comprehensive view of the examination scope of Public Health work. Sanitary law has received somewhat full treatment, each subject being accompanied by its appropriate legal provisions, so that the reader may readily grasp the practical application of the law in administration.

The choice of subjects and their treatment is, as a rule, good; but we miss any consideration of food and food values, though the law relating to food is dealt with at some length, and the housing question is scarcely touched. We venture to think that room might be found in a future edition by condensation in certain of the earlier chapters, in which also some of the illustrations are suggestive of the maker's catalogue, and might well be omitted. The Scottish Acts receive due consideration, as well as the English Public Health Acts, which gives the book a wider usefulness though adding somewhat to its bulk. It is well printed and neatly bound.

**Public Health.** (Catechism Series.) Revised by W. Robertson, M.D., D.P.H. Second Edition. Edinburgh: E. and S. Livingstone. (n.d.)—These five paper-covered volumes of some fifty pages apiece contain, in the form of question and answer, all that the student is expected to know about Public Health at the time of his examination, and all that he is certain to forget immediately afterwards if he relies on this method of preparing himself for that ordeal. The fact that the books are revised by the well-known Medical Officer of the Port of Leith is a guarantee that the answers are correct and well constructed to satisfy the examiners, but we doubt whether the time has not come at which Public Health should cease to be regarded as a troublesome adjunct to the final professional examination to be "crammed" up in the shortest possible time by means of a catechism of this kind. For those students who have given the requisite time and attention to the subject, a few test papers will provide all the experience necessary before entering the examination room; for those who are in a contrary case we do not think that cram books should be provided, and the fact that such books are carefully prepared does not, in our opinion, do anything towards further justifying their existence.

**A Code of Rules for the Prevention of Infectious and Contagious Diseases in Schools.** Sixth Edition. Pp. 64. London: J. & A. Churchill. 1910.—This useful and well-known publication, issued by the Medical Officers of Schools Association, deals with the whole subject of preventive medicine in schools as regards epidemic disease. Important alterations occur in the present edition in the periods of quarantine and isolation assigned to several diseases, mainly in the direction of shortening them. The rules will no doubt continue, as heretofore, to be regarded as authoritative in the matters with which they deal, and will be adopted by all who have medical care of boys and girls in secondary schools, whether boarding schools or day schools.

**Dispensing made Easy.** By Wm. G. Sutherland, M.B. Fourth Edition. Revised by F. J. Warwick, M.B. Pp. vii., 102. Bristol: John Wright & Sons Ltd. 1910.—This little book cannot fail to be of service to those who have not yet learnt, but are still required, to dispense for themselves. "It is only by the adoption of systematic methods, by the use of concentrated solutions of solid salts and other time-saving devices and wrinkles, that anything approaching accuracy combined with rapidity can be obtained." In busy club practice rapid dispensing is essential, and this book gives many useful hints showing how this irksome and tedious branch of the general practitioner's duties may best be accomplished. The use of tablets, coated pills and concentrated mixtures has greatly simplified the art of dispensing, but economy as well as rapidity in dispensing is still necessary in club and parish practice. We commend this book warmly to those who have not established a system of their own in dispensing.

**The Treatment of Syphilis by the Ehrlich-Hata Remedy (Dioxydiamido-arsenobenzol).** By Dr. Johannes Bresler. Translated by Dr. M. D. Eder. Pp. xii, 123. London: Rebman Limited. 1910.—This is a valuable little brochure embodying a collection of observations on the Ehrlich-Hata antisyphilitic remedy, sometimes designated "606," published in various periodicals. Many different methods of employing the drug are described, and results gratifying or unpleasant are freely set forth. The diverse manifestations of syphilis which have been reported as treated by this remedy are briefly recorded. To any one contemplating the adoption of "606" as a treatment in any particular case the information this pamphlet contains will prove most helpful. Facts only are recorded, without special pleading, and almost without critical comment. The reader is given certain data, from which according to his ability he must form his own judgment. This is fair dealing with a drug that has suffered much at the hands of over-zealous advocates.

**Annual Report of the Smithsonian Institution. 1909.**—The high standard of interest for which the Smithsonian Reports are so famous is well maintained in the volume for 1909, which contains a wonderful collection of papers describing the progress of modern science in many of its most important branches. The importance of electricity gives this force a prominent position in no less than four of the contained articles, namely: Radiotelegraphy, by Fleming; Progress in Physics, by Thomson; Conservation of Natural Resources, by Douglas; Nitrogen Question, by Munroe. Two interesting accounts of Antarctic discoveries occur side by side—one by Maurice Zimmermann, the other by Shackleton—and as these have been written from the scientific point of view they are comparatively free from the description of privations and horrors by which similar accounts are often marred. "Charles Darwin" is the title of a very readable lecture by August Weismann, and the far-reaching effect of the teaching of evolution is put lucidly and impressively. The author begins by saying that the influence of Darwin's theories on his time and the future cannot be fully appreciated by any person who has not first brought himself to realise the position of science in 1859. Biology, so to speak, sprang into existence as a coherent science only when zoology, botany, and anthropology were linked together by the theory of evolution. Such sciences as ontogenesis, physiology, and anthropology by the light of evolution were completely changed; in fact evolution shed quite a new light on all the world, both organic and inorganic, and in practical politics this light is now helping to solve the problem of the development of "classes" and their union in the state. A paper by G. Grant MacCurdy, of the Yale University, on the "Antiquity of Man" is especially worthy of notice. It is very exhaustive, and is so written as to present to the reader a varied picture of the life habits of our forbears. The volume, in fact, is replete from cover to cover with interesting facts expressed in the most fascinating manner.

**Merck's Annual Report.** Vol. XXIII. 1909. Darmstadt: E. Merck. July, 1910. London: 16 Jewry Street, E.C.—This brief and impartial review of the therapeutic acquisitions of the last twelve months has now become a hardy annual, much appreciated by those who read it, and of much value as an occasional book of reference. It contains an excellent résumé of the recent advances in pharmaceutical chemistry and therapeutics, not less than eighty-four pages being devoted to the subject of serum therapy and bacterio-therapeutic preparations. A copy of the book can be obtained by any medical man on application to the London office. We advise all who have not done so to secure a copy and study it. It is interesting to know the names and properties of many new drugs, even if we may not have the opportunity of verifying what is said about them before they become replaced by yet newer ones.

**Index-Catalogue of the Library of the Surgeon-General's Office, United States Army.** Second Series. Vol. XV. S.—Skin-grafting. Pp. 777. Washington: Government Printing Office. 1910.—The present volume of this most useful publication includes 804 author titles, representing 4,688 volumes and 7,460 pamphlets, in addition to 3,616 subject titles of books and pamphlets, and 28,328 titles of articles in periodicals. As an indication of the way in which recent medical literature has grown, and the need of such a work as this, we may mention that there are sixteen closely-worded pages of references to "serodiagnosis" and "serotherapy," and about nineteen more pages of references devoted to "serum," "serum-agglutinins," "serum-hæmolysis," etc. Each volume of the Index-Catalogue increases our admiration for those responsible for its production, and lays the profession under a further debt of obligation.

